# Human papillomavirus-associated rectal adenocarcinoma

**DOI:** 10.1093/jscr/rjag017

**Published:** 2026-01-30

**Authors:** Andrew T Myers, Suzan A Bilgesu

**Affiliations:** Division of General Surgery, Geisinger Medical Center, 100 N. Academy Ave, Danville, PA 17822, United States; Division of Colorectal Surgery, Geisinger Medical Center, 100 N. Academy Ave, Danville, PA 17822, United States; Geisinger Commonwealth School of Medicine, 525 Pine Street, Scranton, PA 18509, United States

**Keywords:** rectal adenocarcinoma, human papillomavirus (HPV), p16 protein, total neoadjuvant chemotherapy and radiation (TNT), complete clinical response (CCR)

## Abstract

Human papillomavirus (HPV)-mediated rectal adenocarcinoma is a rare variant of colorectal adenocarcinoma that has only been described in a few case reports and series, with most originating from South America and the Middle East. This report, from North America, details the presentation, workup, and management of a 59-year-old Caucasian female who initially presented to her primary care with rectal bleeding who underwent interval colonoscopy and was found to have a T2 or early T3 rectal adenocarcinoma 1.2 cm from her anal verge. The tumor’s unique pathologic feature demonstrated adenocarcinoma strongly positive for p16 protein, suggesting mediation by HPV. She is currently undergoing total neoadjuvant chemotherapy and radiation for this pathology-proven, HPV-mediated low rectal adenocarcinoma. Based on a thorough literature review, she is likely one of the first patients with HPV-mediated adenocarcinoma to be managed with total neoadjuvant chemotherapy and radiation with hopes for a complete clinical response.

## Introduction

Rectal cancer is the eighth most common cancer worldwide, and there are about 46 000 new rectal cancer diagnoses in the USA every year [1, 2]. Human papillomavirus (HPV) is an oncovirus that is well known to mediate anogenital malignancies, but there have been fewer than 300 cases of rectal adenocarcinoma associated with HPV reported in literature [3, 4]. The standard of treatment for locally advanced rectal cancers in the USA for much of the 21st century has been preoperative pelvic radiation plus chemotherapy total neoadjuvant chemotherapy and radiation (TNT) followed by surgery [5–7]. More recent rectal cancer trials have employed a watch-and-wait strategy following complete clinical response (CCR) to TNT, with a goal to avoid surgical intervention in patients who are willing to undergo rigorous surveillance. This strategy has been shown to be noninferior for local recurrence and overall survival at almost 3 years of follow-up [8, 9]. However, none of these trials explicitly state if the included patients’ adenocarcinomas were HPV-mediated. We present a 59-year-old patient with an HPV-mediated primary rectal adenocarcinoma currently undergoing TNT who strongly desires to avoid surgical management of her disease.

## Case report

The patient is a 59-year-old female with a medical history of chronic ischemic left middle cerebral artery stroke on aspirin and clopidogrel, hypertension, hyperlipidemia, protein-S deficiency, and a prior Papanicolaou (PAP) smear with a low-grade squamous intraepithelial lesion who presented to the emergency department with 1 week of rectal bleeding after a bowel movement. She was diagnosed with bleeding internal hemorrhoids and discharged with steroid cream and stool softeners. Coincidentally, she had routine gynecology follow-up, and a PAP smear was obtained, and the results were negative for intraepithelial lesion and malignancy. The patient presented 2 months later with recurrent rectal bleeding at which time a computed tomography (CT) abdomen and pelvis was obtained and demonstrated diverticulosis. She underwent colonoscopy 1 month later, and a sessile, non-obstructing mass was found in the rectum and biopsied. The pathology report demonstrated a traditional serrated adenoma though a comment in the report stated, “an underlying invasive component cannot be completely excluded, especially in light of the endoscopic findings.” Thus, she underwent repeat endoscopy and had the area in question removed en bloc with endoscopic submucosal dissection (ESD). It was noted to be near the anal verge, 12 mm in maximum thickness, and involved 40% of the lumen of the colon. Endoscopic ultrasound suggested invasion into the submucosa. A biopsy of the resection margin was also obtained. The pathology of the ESD was HPV-associated adenocarcinoma, and the deep margin biopsy demonstrated the same. Next-generation genomic sequencing was significant for a Kirsten rat sarcoma viral oncogene homolog (KRAS) mutation; carcinoembryonic antigen level was 1.7. A CT of the chest/abdomen/pelvis did not demonstrate distant metastasis. An aborted magnetic resonance imaging (MRI) of the rectum secondary to retained biopsy clips prompted a flexible sigmoidoscopy for clip removal; during this procedure, an additional biopsy was obtained, and the pathologist reported that the tumor cells were strongly positive for p16 protein. A subsequent rectal cancer protocol MRI found the tumor to be 1.2 cm from the anal verge and involved the internal anal sphincter. Her case was discussed at multidisciplinary tumor board, and she met with colorectal surgery and radiation oncology physicians. The patient strongly desired to avoid surgery, so according to standard treatment guidelines, TNT was proposed. Given the tumor had sphincter involvement, abdominoperineal resection was discussed. She completed radiation treatment with a total of 5400 cGy with concurrent capecitabine (1500 mg PO BID M–F) for the duration of her radiation. Then, after a 4-week interval, she began neoadjuvant CapeOX [capecitabine 850 mg/m^2^ PO BID Days 1–14 + oxaliplatin 130 mg/m^2^ intravenous (IV) q21 Days (6 cycles)]. She has intermittent peripheral neuropathy and intolerance to cold liquids, but she continues to work full-time. She anticipates finishing her TNT in February 2026 at which time she plans to undergo repeat flexible sigmoidoscopy to evaluate her clinical response.

## Discussion

In addition to highlighting the importance of prompt and thorough evaluation of hematochezia, this case advances the application of the ever evolving and multidisciplinary management of locally advanced rectal cancers. The well-documented benefits of TNT therapy have percolated their way into management of locally advanced rectal cancer, and TNT has become the standard of care. Trials such as RAPIDO and PRODIGE 23 have laid the groundwork for improvements in pathologic complete response rates and 3-year disease-free survival [7, 10].

Our patient thus far has followed the RAPIDO protocol. Despite only being a T3 tumor on MRI, her tumor is considered high risk given its HPV genetics. Though HPV-mediated rectal adenocarcinoma was not one of RAPIDO’s inclusion criteria as a high-risk feature, our patient’s unique presentation calls attention to the need for a case-by-case discussion in a multi-disciplinary setting.

As the era of organ preservation in rectal cancer care is ushered in, our patient’s personal preferences correspond with the overarching themes of many landmark “Watch and Wait” (WW) trials. Most local recurrences occur early in the WW period; nearly 99% of recurrences happen within the first 3 years [9]. Intensive follow-up is a must. Our patient remains steadfast in her commitment to following up at the end of her TNT. She is hoping to avoid surgery if at all possible; however, she recognizes that a CCR is not guaranteed. It is yet to be determined if having rectal adenocarcinoma mediated by high-risk HPV will impact her clinical response to TNT. Neither the International Watch & Wait Database (IWWD) registry studies nor the Organ Preservation in Patients with Rectal Adenocarcinoma (OPRA) trial specifically report HPV-associated rectal cancers in their inclusion criteria [9, 11].

Available literature on the few cases of HPV-associated adenocarcinomas does not suggest that it is more aggressive than non-HPV-mediated adenocarcinoma [3]. Rates of both local recurrence and tumor-related death for HPV-mediated adenocarcinomas are similar to non-HPV rectal adenocarcinoma. It is reasonable to consider a WW surveillance program should she have a CCR. The applicability of this patient’s case to others is most definitely limited as it is a single case report and should only be performed after cautious consideration. Yet, it highlights the potential for broadening the application of organ preservation management strategies in a unique genetic subset of rectal adenocarcinomas.
